# Biological Properties of Sandalwood Oil and Microbial Synthesis of Its Major Sesquiterpenoids

**DOI:** 10.3390/biom14080971

**Published:** 2024-08-08

**Authors:** Xiaoguang Yan, Sichone Daniel David, Guangzhao Du, Weiguo Li, Dongmei Liang, Shengxin Nie, Mingyue Ge, Chen Wang, Jianjun Qiao, Yanni Li, Qinggele Caiyin

**Affiliations:** 1 School of Chemical Engineering and Technology, Tianjin University, Tianjin 300072, China; 2 Zhejiang Institute, Tianjin University, Shaoxing 312300, China; 3 Zhejiang Key Laboratory of Alternative Technologies for Fine Chemicals Process, Shaoxing University, Shaoxing 312000, China; 4 Key Laboratory of Systems Bioengineering, Ministry of Education, Tianjin University, Tianjin 300072, China

**Keywords:** sandalwood oil, sesquiterpenes, santalene, santalol, biosynthesis, metabolic engineering

## Abstract

Sandalwood essential oil is extracted from the heartwood part of mature sandalwood and is known for its pleasant fragrance and exceptional medicinal activities, including antimicrobial, antitumor, and anti-inflammatory properties. The (Z)-α-santalol and (Z)-β-santalol are the most vital ingredients contributing to sandalwood oil’s bioactivities and unique woody odor characteristics. Metabolic engineering strategies have shown promise in transforming microorganisms such as yeast and bacteria into effective cell factories for enhancing the production of vital sesquiterpenes (santalene and santalol) found in sandalwood oil. This review aims to summarize sources of sandalwood oil, its components/ingredients, and its applications. It also highlights the biosynthesis of santalene and santalol and the various metabolic engineering strategies employed to reconstruct and enhance santalene and santalol biosynthesis pathways in heterologous hosts.

## 1. Introduction

Plant essential oils are complex mixtures of various low molecular weights with aromatic volatile compounds. They are oleaginous and frequently characterized by a strong scent [1]. Plant essential oils are regularly distributed in plant parts such as leaves, flowers, seeds, bark, wood, fruits, roots, etc., and are usually obtained through various methods, including extraction, expression, or fermentation. However, steam distillation is widely used to commercialize essential oils [2,3]. Plant essential oils consist mainly of volatile compounds, including terpenes. Over time, these oils have been widely used in ethnomedicine and possess fascinating biological effects, making them potential substitutes for traditional medications [4]. However, several essential oils have been shown to possess multiple functional properties beyond their conventional roles. This is because several biological agents have evidenced the antimicrobial properties of essential oils, encompassing antibacterial, antifungal, and antiviral effects, along with anti-inflammatory and anticancer activities [1,5,6,7]. Apart from that, the essential oils derived from plants, such as sandalwood oil, form a significant and crucial component of odorants used in various commercial applications, especially in the perfumery or cosmetics industries [8,9].

Traditional methods, reliant on direct plant extraction, are time-consuming, land-intensive, and costly due to low yields and high production costs. Especially for the sandalwood oil, the limited sandalwood resources restrict their production. In contrast, microbial biosynthesis enables the production of plant natural products through fermentation using renewable resources, leading to cost-effective and sustainable production processes and offering significant economic and environmental advantages. Driven by technological progress in metabolic engineering, process biotechnology, and synthetic biology, terpene-based bioactive compounds such as medicines, food ingredients, and perfumes produced by microbial fermentation have become viable and commercially competitive options [10,11,12]. Metabolic pathway design and enzyme engineering reform are key points for improving cell factory performance [13,14].

This review provides a general understanding of sandalwood essential oil and the microbial synthesis of its major terpenoids, specifically focusing on recent progress in metabolic engineering strategies to increase the santalene and santalol yield in engineered microbial cells.

## 2. The Source of Sandalwood Essential Oil, Its Main Components, and Its Applications

**Figure 1:**
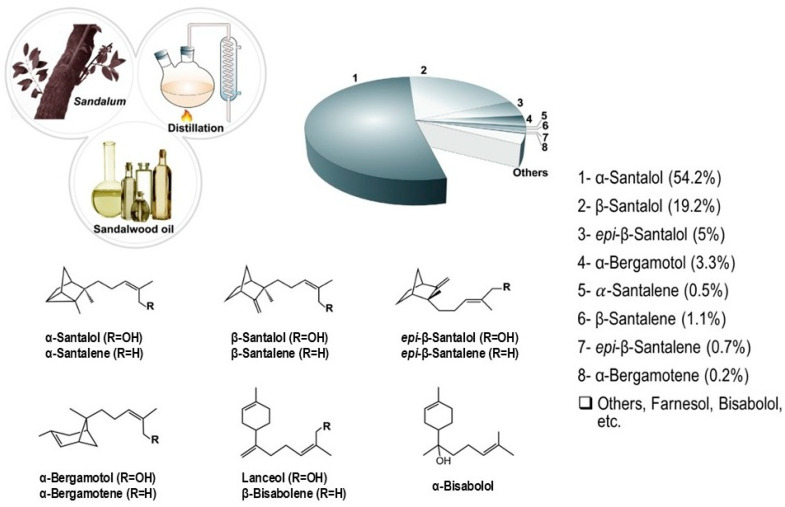
Percentage composition of terpenoids in sandalwood essential oil [35].

Sandalwood oil is a highly prized plant essential oil primarily obtained from Santalum species such as *S. album*, *S. austrocaledonicum*, *S. yasi*, and *S. spicatum*. It is mainly extracted from the heartwood of a well-matured sandalwood tree through the steam distillation process [15,16]. Other methods have also been used to extract sandalwood oil from the heartwood, including solvent extraction, hydro-distillation, liquid CO_2_ extraction, and supercritical fluid extraction (SC-CO_2_) [17]. The amount, composition, and physiochemical characteristics of sandalwood essential oil depend on the species of origin, the tree’s age, the region where it is grown, the season of harvest, and the extraction method used [9]. The main compositions of sandalwood essential oil are sesquiterpenoids ((Z)-α- santalol, (Z)-β-santalol, (Z)-α-*exo*-bergamotol, (Z)-*epi*-β-santalol) and sesquiterpenes (Figure 1) (α-santalene, β-santalene, *epi*-β-santalene, and α-*exo*-bergamotene) [18,19,20].

Santalol makes up about 90% of the sesquiterpenoid content in the oil (Table 1); it is composed of about 40–60% α-santalol and from 20 to 25% β-santalol [9]. In general, sandalwood essential oil shall have (Z)-α-santalol content ranging from 41–55% and (Z)-β-santalol in the range of 16–24% [18]. However, at present, *S. spicatum* extracts do not meet that requirement for two main reasons: a low combined santalol content and high levels of E-farnesol isomer, a suspected allergen [21,22]. Meanwhile, the Indian native sandalwood tree species *S. album* is historically said to provide the bulk of sandalwood products, which contain a higher oil content rich in santalol (approximately 90%) [23]. Extracts of *S. spicatum* are typically less valuable than those of *S. album* due to the lower total santalol content and diverse sesquiterpene composition [24].

The (Z)-α-santalol and (Z)-β-santalol are the most vital ingredients contributing to sandalwood oil’s bioactivities and unique woody odor characteristics. The double-bond configuration of the α-santalol molecule plays a crucial role in determining its odor [25]. The presence of a high content of santalol and santalene makes sandalwood oil possess several pharmacological/medicinal activities, such as anticancer activity [26,27,28], antimicrobial activity [29,30], antiviral activity [31,32], and neuroleptic/antipsychotic activity [33]. Preclinical and clinical research has indicated that sandalwood extracts have shown anticancer and antimicrobial activities and suggests that they possess antioxidant, anti-inflammatory, antihyperglycemic, and antihyperlipidemic activity [34].

### 2.1. Antioxidant Properties of Sandalwood Oil

Oxidative stress is known to impact the onset of various diseases significantly. Throughout history, the effectiveness of sandalwood oil’s antioxidant properties in counteracting damage caused by oxidative stress has been widely acknowledged. Numerous studies have demonstrated that Indian sandalwood oil possesses noteworthy antioxidant capabilities, effectively neutralizing reactive oxygen species triggered by external stressors such as urban dust, exposure to blue light, and inhalation of cigarette smoke [36,37]. Sandalwood oil has been shown to reduce levels of squalene monohydroperoxide, an indicator of oxidative stress, in a dose-dependent manner. Concentrations ranging from 1% to 10% have demonstrated protective properties against oxidative stress caused by exposure to urban dust and blue light [36]. Furthermore, the antioxidative efficacy of sandalwood oil has been validated through a variety of in vitro studies, demonstrating its ability to counteract free radicals and prevent the escalation of collagenase levels caused by pollutants, highlighting its potential as a protective and anti-aging component in the fields of cosmetics and dermatology [37]. In in vivo oxidative stress-induced models, α-santalol and sandalwood oil would modulate parameters such as serum aminotransferases, alkaline phosphatase, bilirubin, superoxide dismutase, catalase, free sulfhydryl, protein carbonyl, nitric oxide, and liver lipid peroxide contents, which directly showed their antioxidant properties [38]. Subsequent research has revealed that sandalwood essence has phytoconstituents with potent antioxidant properties, making it a suitable candidate for use in the pharmaceutical and food industries [39].

### 2.2. Cytotoxicity of Santalol

Santalol, derived from sandalwood essential oil, is being studied for its anticancer activity; its ability to suppress, delay, or even reverse the carcinogenesis process makes it one of the best chemoprevention agents [26]. Chemoprevention is the control of cancer using natural or synthetic agents, either by blocking (preventing carcinogenic substances from reaching critical target sites during the initiation stage of cancer development) or suppressing (inhibiting the proliferation of malignant cells during the promotion and development of carcinogenesis) [40]. α-Santalol demonstrates antitumor properties by inhibiting the growth and viability of malignant cells. This impact extends to various cancer cell types, including breast cancer cells, regardless of their estrogen receptors and p53 status [41]. Additionally, α-santalol affected the localization of β-catenin, one of which is the major signaling pathways associated with cancer development and metastasis, thus inhibiting the migration of breast cancer cells [28]. Moreover, α-santalol acts as a chemopreventive agent against UVB-induced skin tumor development by inhibiting inflammation and epidermal cell proliferation and promoting apoptosis [42].

### 2.3. Sedative and Anxiolytic Effects of Sandalwood Oil

Sandalwood oil has sedative and anxiolytic properties and serves protective functions against cerebral damage by inhibiting oxidative stress and inflammatory pathways [43]. The α-santalol has been shown to exhibit anxiolytic-like action, lowering locomotor activity following stress exposure. It can also transfer to the brain, adding to its calming effects [44]. Sandalwood oil has also been used to treat neurological complications by influencing neurotransmitter systems such as gamma-aminobutyric acid (GABA), dopamine, serotonin, and acetylcholine, which can regulate physiological effects in the brain and possibly enhance memory function [45]. Also, santalol exhibits neurotherapeutic activities and psychotropic drug characteristics. Research indicates that when inhaled at specific concentrations, santalol can significantly impact the sleep-wake cycle by decreasing total waking time and increasing non-rapid eye movement sleep time [46]. Moreover, sandalwood extracts are essential to the fragrance industry due to their warm, woody scent. They are used in various cosmetics, fragrances, flavors, and pharmaceutical industries [16,30,47].

## 3. Biosynthesis of Santalene, Santalol, and Other Terpenoids Found in Sandalwood Oil

**Figure 2:**
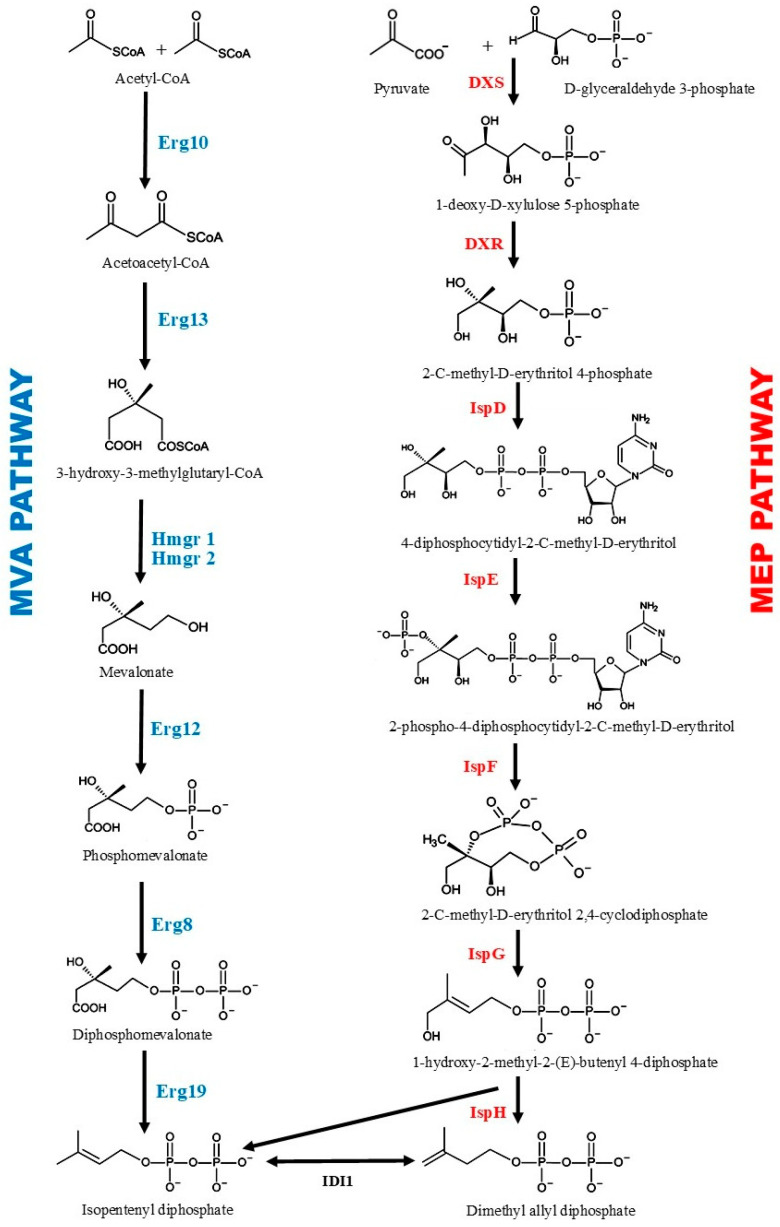
Isoprenoid biosynthesis pathway. The MVA pathway (**Left**). Enzymes: Erg10, acetoacetyl-CoA thiolase; Erg13, 3-hydroxy-3-methylglutaryl-CoA synthase; Hmg1/2, 3-hydroxy-3-methylglutaryl-CoA reductase; Erg12, mevalonate kinase; Erg8, phosphomevalonate kinase; Erg19, mevalonate diphosphate decarboxylase; Idi, isopentenyl diphosphate isomerase. The MEP pathway (**Right**). Enzymes: Dxs, 1-deoxy-D-xylulose-5-phosphate synthase; Dxr, 1-deoxy-D-xylulose 5-phosphate reductoisomerase; IspD, 4-diphosphocytidyl-2-C-methyl-D-erythritol synthase; IspE, 4-diphosphocytidyl-2-C-methylerythritol kinase; IspF, 2-C-methyl-D-erythritol 2,4-cyclodiphosphate synthase; IspG, 4-hydroxy-3-methylbut-2-en-1-yl diphosphate synthase; IspH, 1-hydroxy-2-methyl-butenyl 4-diphosphate reductase.

Despite the vast structural diversity of terpenoids, they all originated from two 5-C molecules, namely, isopentenyl diphosphate (IPP) and dimethylallyl diphosphate (DMAPP), which are naturally produced via the mevalonic acid (MVA) pathway and non-mevalonic acid pathway also known as 2-C-methyl-D-erythritol-4-phosphate (MEP) pathway (Figure 2) [48,49,50]. The MVA pathway, native to plant cytosol and eukaryotes, has been successfully integrated into yeast [51,52] and bacteria [53]. Six enzymic catalytic steps (Table 2) direct carbon flux from acetyl-CoA toward IPP formation. Several studies have shown that the enzymes 3-hydroxy-3-methylglutaryl-CoA reductase (HMGR), mevalonate kinase (MK), phosphomevalonate kinase (PMK), and isopentenyl diphosphate isomerase (IDI) are considered to be rate-limiting steps [54,55]. Conversely, a non-mevalonic acid or MEP pathway is native to plant plastids and most eubacteria. Seven enzymic catalytic steps (Table 2) are involved in the formation of IPP and DMAPP from pyruvate and glyceraldehyde-3-phosphate (G3P), whereby it has been shown that four enzymes, 1-Deoxy-D-xylulose-5-phosphate reductoisomerase (DXR), 2-C-methyl-D-erythritol 4-phosphate cytidylyltransferase (ispD), 2-C-methyl-ᴅ-erythritol-2,4 cyclodiphosphate (ispF), and isopentenyl diphosphate isomerase (IDI), are involved in what is known as the rate-limiting step in *E. coli* [56,57].

Also, the isopentenol utilization pathway (IUP) has recently become an alternative metabolic pathway to increase the supply of DMAPP or IPP in recombinant strains. This pathway comprises promiscuous kinase and isopentenyl phosphate kinase, which consecutively phosphorylate prenol into DMAPP via the intermediate dimethylallyl phosphate (DMAP). Promiscuous kinases can be combined with isopentenyl phosphate kinases to provide a simple and effective enzymatic approach for manufacturing DMAPP in vitro, employing prenol as a suitable substrate and ATP as a cofactor [58].

The synthesis of santalene and santalol requires the utilization of FPP as a central building block, serving as the fundamental precursor for all sesquiterpenes. Farnesyl diphosphate (FPP) is created through successive prenylation processes, wherein a DMAPP carbocation initially combines with one IPP unit via an electrophilic alkylation reaction and is subsequently deprotonated to yield GPP—the addition of another IPP unit to GPP results in FPP formation whereby the enzyme farnesyl diphosphate synthase catalyzes these prenylation reactions [59]. The santalene synthases accomplish the conversion of FPP into santalene [59,60]. Multiple isoenzymes of santalene/bergamotene synthase (STS) have been characterized from *Santalum* species [61] and other plant species like *Clausena lansium* [12,62], as well as a camphor tree, *Cinnamomum camphora* [63]. In *Santalum* species, santalene/bergamotene synthases play a crucial role in converting (E, E)-FPP into a mixture of sesquiterpenes, referring to α and β santalene, *epi*-β-santalene, and α-*exo*-bergamotene [60,61,64]. On the other hand, ten cytochrome P450 enzymes from the CYP76F subfamily are essential in converting these sesquiterpenes into corresponding sesquiterpene alcohols, referring to santalol and bergamotol [65,66]. Nine functionally characterized SaCYP76F enzymes belonging to the CYP76F subfamily have been identified as primarily producing (E)-α-santalol, (E)-β-santalol, (E)-*epi*-β-santalol, and (E)-α-*exo*-bergamotol, with a lesser amount of (Z) stereoisomers which are responsible for the aromatic flavor of sandalwood oil [65]. Among these enzymes, the CYP76F39v1 variant demonstrated the highest catalytic efficiency in producing (E) stereoisomers [67]. Recent research has shown that (Z)-α/β-santalol and (Z)-α-*exo*-bergamotol can be selectively synthesized in vitro by the CYP736A167 enzyme from *S. album* [12].

## 4. Enzyme Engineering to Improve Catalytic Properties

The catalytic efficiency of natural sesquiterpene synthases is generally low, affecting sesquiterpenoids’ biosynthetic efficiency. For enzyme engineering, undertaking the following four areas of mutations may enhance the catalytic efficiency of sesquiterpene synthases: (1) explicitly modifying conserved structural domains, (2) aligning active site residues of sesquiterpene synthases, (3) analyzing a multitude of homologous gene sequences followed by selecting the most conserved residues as candidates for targeted mutations, and (4) combining error-prone PCR with high-throughput screening. Focusing on the C-terminus active region of α-santalene synthase (CISS), the strain with a single mutation ClSS^S533A^ has been shown to significantly increase the production of α-santalene by 1.7 folds compared to the control strain. The mutation of residue S533 to Ala resulted in the addition of two hydrogen bonds near this site (<4 Å), which resulted in improving the catalytic efficiency of CISS [68]. Fusion protein will reduce the spatial distance between the substrate and the enzyme, thereby enhancing the catalytic efficiency of sesquiterpene synthases. Through expression of the fusion proteins with ERG20 and related terpene synthases, some terpenes, like (+)-Valencene, α-farnesene, and patchoulol, have been synthesized efficiently in microorganisms [69,70,71]. While there are no reports of employing this strategy to enhance the titer of santalene, it is certainly a promising approach for increasing the catalytic efficiency of santalene synthase.

QM/MM computational simulations were employed to obtain the catalytic reaction energy profiles for SaSSy and SanSyn. It was found that T318 in SaSSy and T298 in SanSyn can be involved in the deprotonation of the carbocation intermediate. F441 of SanSyn is seen as a key residue restricting the conformational dynamics of the intermediates, and thereby, the direct deprotonation by the general base T298 dominantly produces α-santalene. The subsequent mutagenesis of this plastic residue leads to generating a mutant enzyme, SanSyn F441V, which can make both α- and β-santalenes. By this method, the enzyme specificity and the plant essential oils with desirable component ratios were obtained by the combination of metabolic and enzymatic engineering [72]. 

Except for sesquiterpene synthases, the engineering of P450 monooxygenases is crucial for enhancing santalol biogenesis. The P450 monooxygenases form a broad and versatile family of oxidative enzymes ubiquitously found within organisms. They are critical in catalyzing most rate-limiting reactions in terpenoid biosynthetic pathways. These enzymes carry out particular oxidation reactions, utilizing their intricate catalytic mechanisms to transform various chemical substances, including drugs, toxins, and endogenous molecules. The P450 enzymes are typically involved in single electron transfer processes, which activate oxygen to generate intermediates, subsequently oxidizing the substrates. The P450 core structure consists of a heme-iron reactive center with the catalytic site around the heme-containing comparatively small peptide regions (termed substrate recognition sites, SRS) involved in substrate binding and catalysis. These SRS show high amino acid sequence variation, underlying the ability of different P450s to bind diverse substrate types [73]. The N-terminus membrane binding region of P450 is also essential for its expression and solubility. Furthermore, the co-expression of a plant CPR, the reductive partner of P450, is required to obtain the high plant P450 activity in heterologous hosts [74]. The low heterologous expression, poor stability, and low catalytic activity of P450 represent the bottlenecks of santalol heterologous biosynthesis. However, there has yet to be a report about the engineering modification of P450 for the formation of santalol. The mutations were introduced at the N-terminal region, which could improve the expression and solubility of P450 [75]. The fusions of some P450 with CPR could increase the oxidation efficiency of the products [76]. Furthermore, enhancing intracellular heme biosynthesis to improve the titers and functional activities of hemoproteins [77], like P450s, could also be an intelligent strategy for efficiently synthesizing terpenoids. The effective combination and application of these strategies may improve the catalytic efficiency of santalol.

## 5. Metabolic Engineering Strategies to Increase the Yield of Santalene and Santalol in *S. cerevisiae*

Metabolic engineering strategies have shown promise in transforming microorganisms such as yeast and bacteria into effective cell factories for enhancing the production of certain chemical substances. As stated earlier, the availability of sandalwood oil intensely depends on extracting it from the heartwood part of sandalwood. The sustainability of natural sources of sandalwood oil, such as *Santalum* species, is uncertain due to overexploitation, long growth time to maturity, and climate changes [78]. Records show that the annual consumption of Indian sandalwood is approximately 4000 tonnes. According to 2004 records, oil production has decreased drastically to around 70 tonnes compared to the last six decades, when the output of Indian sandalwood oil was nearly 180–200 tonnes, sufficient to meet consumer demand. This drastic fall in sandalwood oil production is due to a shortage of sandalwood trees [79]. Overcoming these limitations necessitates adopting a sustainable approach for the large-scale production of santalene and santalol to meet commercial demands [16].

*S. cerevisiae* is among the numerous microorganisms used for centuries to synthesize valuable biomolecules. It is a single-celled eukaryotic microorganism belonging to the fungi kingdom and is the most extensively studied eukaryotic microbe, providing insight into the biology of eukaryotic cells [80]. Due to its non-pathogenic characteristics, *S. cerevisiae* is considered a GRAS organism (Generally Recognized as Safe). *S. cerevisiae* is a valuable asset in biotechnology, offering many possibilities for innovative applications and advancements. It is known for its ability to utilize various carbon sources, ease manipulating sterol pathways by introducing exogenous genes such as TPSs [81,82], robustness, and adaptability to harsh conditions [13]. This makes *S. cerevisiae* an indispensable tool in the biotechnological production of terpenoids.

**Figure 3:**
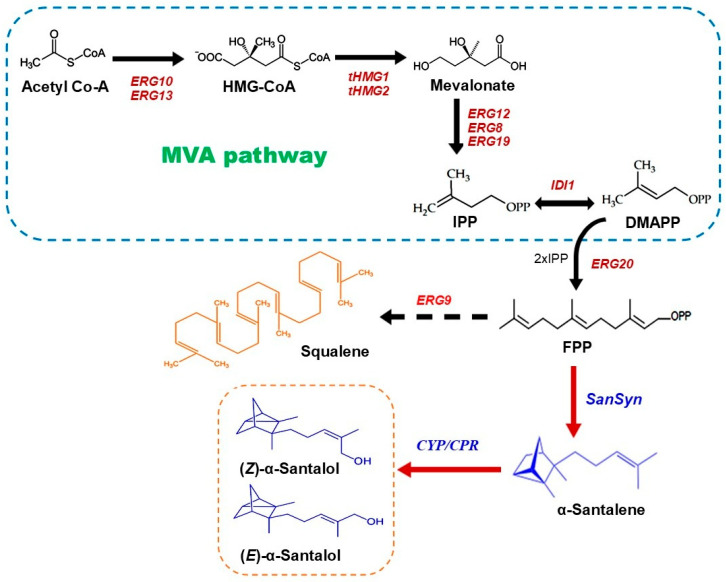
The biosynthetic pathway of α-santalene and α-santalols in *S. cerevisiae*. The black arrows represent the steps catalyzed by native enzymes, and the red arrows represent the steps catalyzed by exogenous enzymes. Meanwhile, the dashed arrow represents a step that is depressed. (ERG10: acetoacetyl-CoA thiolase; ERG13: HMG-CoA synthase; HMG-CoA: 3-hydroxy-3-methyl-gluraryl-CoA; tHMG1 and tHMG2: a truncated HMG-CoA reductase; ERG12: mevalonate-5-kinase; ERG8: phosphomevalonate kinase; ERG19: mevalonate pyrophosphate decarboxylase; ERG20: (E, E)- FPP synthase; ERG9: squalene synthase; SanSyn: α-santalene synthase; CYP: cytochrome P450 monooxygenase; CPR2: NADPH-cytochrome P450 reductase.

In *S. cerevisiae*, the IPP and DAMPP are generated through the MVA pathway. In the first step, acetyl-CoA molecules are transformed into acetoacetyl-CoA catalyzed by acetoacetyl-CoA thiolase enzyme (ERG10). The subsequent condensation of acetoacetyl-CoA with another acetyl-CoA results in the formation of 3-hydroxy-3-methylglutaryl-CoA (HMG-CoA) catalyzed by HMG-CoA synthase (ERG13). Following this, HMG-CoA’s thioester is converted into alcohol by the HMG-CoA reductase enzyme, producing mevalonate. This set of reactions involves two consecutive reduction reaction mechanisms, each demanding one NADPH H^+^ molecule: the first for the conversion of the thio-esterified carboxyl group into an aldehyde and the second for the transformation of the aldehyde into alcohol [83,84,85]. In *S. cerevisiae*, two isoenzymes exist: Hmg1 and Hmg2. These isoenzymes exhibit HMG-CoA reductase activity, with Hmg1p noted for their prominent role in yeast enzyme activity [86]. Both Hmg1 and Hmg2 are located in the endoplasmic reticulum membrane. Then, mevalonate undergoes two consecutive phosphorylation reactions catalyzed by ERG12 and ERG8 to yield mevalonate phosphate and mevalonate diphosphates, respectively, whereby the second phosphorylation reaction is reversible. The subsequent response involves the decarboxylation of mevalonate diphosphate to yield IPP, a reaction catalyzed by ERG19, which is ATP-dependent. Lastly, DMAPP is generated through an isomerization reaction of IPP by isopentenyl diphosphate isomerase (IDI1) [87]. The santalenes and santalols can be obtained by reconstructing the biosynthetic pathway of santalenes and santalols in *S. cerevisiae* through the introduction of exogenous genes such as STSs, which converts FPP into santalenes and CYPs, alongside its redox partner cytochrome P450 reductases (CPRs), which oxidizes santalenes into santalols (Figure 3). The following metabolic engineering strategies have been employed to increase the yield of santalene and santalol in *S. cerevisiae*.

### 5.1. Engineering of the Key Enzymes in the MVA Pathway

To increase the supply of IPP and DMAPP, efforts have been made to manipulate the MVA pathway in *S. cerevisiae* (Table 3). This involves overexpression and increasing the transcript levels of key genes in the MVA pathway. The conversion of HMG-CoA to mevalonate is a crucial step in this context; HMGR is a highly regulated enzyme recognized as responsible for controlling the flow in the MVA pathway [88]. Also, HMG-CoA reductase is an important enzyme that regulates sterol synthesis by interacting with sterol trigger sensors of the endoplasmic reticulum membrane, eliminating the sterol regulation feedback. This is achieved by expressing an improved form of HMGR, a truncated (tHmg1) lacking the NH2-terminal. Overexpression of the HMG1 has been successfully used earlier and revealed to have the most significant impact on producing alcohols in *S. cerevisiae* via the MVA pathway [89,90]. 

ERG20 catalyzes the condensation reaction of IPP to form GPP and FPP. It has been identified that converting IPP to FPP in *S. cerevisiae* is a flux-regulatory step that is firmly controlled in the MVA pathway [94,95]. FPP is a universal precursor not limited only to sesquiterpenes but also acts as an intermediate substance for many other compounds, including ergosterol, ubiquinone, etc., where the overexpression effectiveness of the ERG20 gene to maximize the FPP yield depends on the yeast strain used and culturing conditions [96,97]. It has been shown that integrating and over-expressing the genes glutamate dehydrogenase GDH2 and ERG20 significantly improved sesquiterpene synthesis, subsequently maximizing the α-santalene yield. Also, the α-santalene yield was improved upon further up-regulation of these two genes [88]. Furthermore, integrating and over-expressing the mutant transcription factor gene and an additional supply of truncated HMG1 into the yeast genome improves the effectiveness of the MVA pathway [52]. 

### 5.2. Engineering of FPP Branch Point

**Figure 4:**
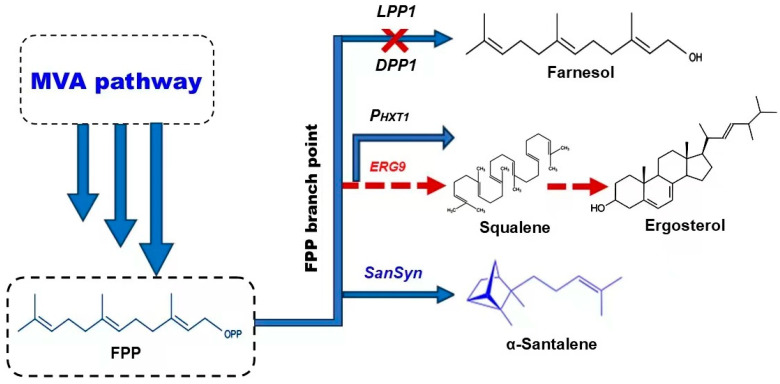
Schematic diagram showing FPP branch point. To increase the FPP pool towards santalene synthesis, ERG9, the encoding squalene synthase, is downregulated by replacing its promotor with PHXT1, and the lipid phosphate phosphatases LPP1 and DPP1, enhancing the FOH formation, are deleted. FPP: farnesyl diphosphate; FOH: farnesol; SanSyn: santalene synthases.

Overexpressing key MVA pathway genes is expected to expand the pool of FPP and enhance the flow of FPP into other pathways, such as the synthesis of sterols from squalane as a precursor, which is formed through condensation of FPP molecules under squalane synthases, encoded by ERG9 genes (Figure 4) [96]. Due to yeast cells’ elevated sterol requirements, ergosterol biosynthesis exhibits high activity levels, leading to the predominant consumption of most FPP [98,99]. Consequently, manipulating the FPP branch point represents a crucial approach to guiding the flux toward α-santalene production while reducing the excess FPP directed toward squalane generation. Several gene promoters have depressed the squalene synthase expression, redirecting FPP flux toward sesquiterpene synthesis. Substituting the strong promotor ERG9 with a weak one, such as MET3 and CTR3, has efficiently inhibited ergosterol biosynthesis [10,100,101]. The glucose-induced promoter, PHXT1, is predominantly activated at elevated glucose levels in *S. cerevisiae*. It has been observed to exhibit greater efficacy in suppressing the expression of ERG9 in yeast, thus leading to an increase in α-santalene production [88,89]. Also, recent research achieved a high titer of santalene and santalol by depressing ERG9 gene expression in *S. cerevisiae* [12]; it was also deduced that downregulating ERG9 enhanced target product production while decreasing by-product output, with PHXT1 being more effective than PERG1 [67]. The Farnesol biosynthesis pathway from the FPP branch involves two phosphatase genes, LPP1 and DPP1, which are lipid phosphate phosphatase codes [102]. Down-regulation of DPP1 led to a significant improvement in santalene synthesis and suppression of farnesol biosynthesis in *S. cerevisiae* [88,89]. The high yield of other terpene products like patchoulol and *trans*-nerolidol was also achieved through manipulating the FPP branch point [70,103].

### 5.3. Manipulation of NADH and NADPH Cofactors

NADPH is a crucial enzymatic cofactor essential for numerous cellular processes. Both the enzyme tHMG1 and the Cytochrome P450 enzymes (CYPs) rely on the presence of NADPH to carry out their biological functions, and as a result, increasing the levels of NADPH within the cell can lead to improved terpenoid biosynthesis in the yeast species *S. cerevisiae* [104]. The process that generates α-santalene involves the overall generation of NADH and the utilization of NADPH. Manipulating the equilibrium of NADH and NADPH co-factors to address restrictions imposed by cellular redox conditions is a widely recognized approach in metabolic engineering to improve the synthesis of sesquiterpenes [105]. Increasing NADPH through altering the ratio of NADH and NADPH gives more advantages to product production. Likewise, enhancing the supply of the NADPH-reduced cofactor by removing the NADPH-consuming process involving glutamate dehydrogenase, as documented with the deletion of GDH1, has been utilized formerly to improve product yield [106]. Similarly, increasing the expression of GDH2’s NAD-dependent glutamate dehydrogenase activated a distinct ammonium consumption route. This resulted in increased NADH flow during the metabolic construction process and a shift in cofactor balance within the yeast [107]. Scalcinati et al. performed genetic alterations by deleting GDH1 and increasing GDH2 to minimize NADPH use during ammonium absorption. This intervention significantly increased α-santalene synthesis in the engineered strain of *S. cerevisiae* [88]. Also, overexpressing 2,3-butanediol dehydrogenase mutant is one successful strategy for increasing NADPH supply in *S. cerevisiae* [108,109].

### 5.4. Engineering of the Acetyl-CoA Supply

In *S. cerevisiae*, ALD, ACS, and ADH are crucial in the synthesis and regeneration of acetyl-CoA. Pyruvate decarboxylase catalyzes the dehydrogenation of acetaldehyde and the combination of acetic acid and CoA to produce acetyl-CoA. In contrast, ALD and ACS catalyze acetaldehyde dehydrogenation and the reversible conversion of acetaldehyde to ethanol [110]. Overexpression genes encoding acetyl-CoA synthase, such as ACS1 and ACS2, in *S. cerevisiae* improve its physiological functions. It leads to increased intracellular acetyl-CoA and ATP content, upregulation of essential genes that play a crucial role in the MVA pathway for terpene synthesis, and improved tolerance to high ethanol levels [111]. Also, it was discovered that overexpressing ACS2 increases resistance to acetic acid, suggesting that ACS2-facilitated uptake during fermentation leads to the detoxification of acetic acid. To overcome inhibition, overexpressing ACS2 improved the efficiency of *S. cerevisiae* in manufacturing acetyl-CoA from acetic acid [112]. Moreover, it is also shown that a massive amount of acetyl-CoA is used up in cytosol and peroxisome to synthesize citrate and malate through the glyoxylate cycle [113]. Research conducted by Y. Chen et al. discovered that knocking out these two enzymes improved the titer for α-santalene in *S. cerevisiae* [114].

### 5.5. Reconstructing Santalol Biosynthesis Pathway under the GAL Regulatory System

GAL promoters are used to regulate enzyme expression when constructing yeast cell factories. They are flexibly controlled by adjusting galactose levels in *S. cerevisiae* culture [10,52]. A recent study linked santalol’s biosynthetic pathway to the GAL regulatory system as well as the overexpression of critical enzymes in the MVA pathway. Overexpression of a transcriptional activator of GAL genes, such as GAL4 and phosphoglucomutase PGM2, increased galactose uptake, improving α-santalol production in engineered *S. cerevisiae* strains [12]. This highlights the importance of GAL4 and PGM2 in regulating gene expression.

### 5.6. Fermentation Optimization

Fermentation is a crucial process for producing the desired product and substantially impacts the final yield of targeted products in engineered microbial host cells. A study by Tippmann et al. employed fed-batch cultivations with RQ-controlled exponential feeding to increase the production of α-santalene. The exponential feeding in fed-batch cultivations was mentioned to maintain total aerobic respiration while lowering the ethanol build-up [115]. Also, a recent study showed that the yields of santalene and santalol were increased when a fed-batch fermentation approach was used compared to that of flask-based fermentation while carefully regulating the galactose/glucose ratio in the medium [12]. Fermentation optimization strategies may include but are not limited to media optimization (e.g., selection of carbon and nitrogen sources, optimization of pH and temperature conditions, the addition of growth factors and co-factors) and process parameters optimization (e.g., oxygen supply and aeration, agitation, and mixing, fermentation time, and harvesting).

## 6. Conclusions

The characteristics of sandalwood essential oil depend on the species of origin, the seasonal variation, and the extraction methods. Furthermore, the amount, composition, and physiochemical characteristics of sandalwood essential oil will influence its various activities. With the increasing consumption of precious natural resources, the biosynthesis of important components of sandalwood oil in a microbial host is becoming increasingly urgent.

Significant strides have been made in santalene and santalol biosynthesis within microorganisms. Research efforts have primarily concentrated on manipulating microbial strains to boost production levels. These endeavors have involved creating biosynthetic pathways in *S. cerevisiae*, optimizing P450-CPR redox systems, and suppressing the ERG9 gene to enhance the yields of santalene and santalol [67]. Moreover, successful outcomes have been achieved by adjusting the metabolic flux towards sesquiterpenoid synthesis by reducing ERG9 expression and amplifying the expression of santalol biosynthesis genes under GAL promoters in yeast [12]. In the case of *E. coli*, the enhancement of precursor flux and the engineering of santalene synthase have led to a substantial increase in α-santalene production [68]. Additionally, introducing genetic modifications and implementing optimal feeding strategies in *Y. lipolytica* have enabled the heterologous synthesis of α-santalene, resulting in enhanced yields devoid of undesired by-products [91]. These advancements underscore the promising prospects for the efficient biosynthesis of santalene and santalol in microorganisms, paving the way for diverse industrial applications in the future.

Despite this remarkable progress in reconstructing santalene and santalol biosynthesis pathways in microorganisms, some challenges remain. For example, P450-mediated metabolic engineering is often complex due to the limited expression of P450 enzymes. Approximately 40% of plant P450s are poorly expressed or not expressed in yeast and other microbes, which hinders pathway reconstruction in microorganisms [74]. Hence, optimizing the P450/CPR redox system in *S. cerevisiae* for high conversion of α-santalene to α-santalol is considered a bottleneck to santalol heterologous biosynthesis.

## Figures and Tables

**Table 1:** Percentage composition of α-santalol and β-santalol in various Santalum species.

Native/Trade Name	Scientific Name	α-Santalol (%)	β-Santalol (%)
Indian sandalwood	*Santalum album*	41–55	16–24
New Caledonian sandalwood	*Santalum austrocaledonicum*	48–49	20–22
Fiji sandalwood	*Santalum yasi*	37–39	26–28
Australian sandalwood	*Santalum spicatum*	15–25	5–20

**Table 2:** Comparison of biosynthesis pathways for generating terpenoid precursors.

Pathway	Starting Carbon Source	Enzyme Catalyzed Steps	Precursor Formed
MVA pathway	Acetyl-CoA	6	IPP
MEP pathway	Pyruvate and G3P	7	IPP and DMAPP

**Table 3:** Progress in santalene and santalol biosynthesis and metabolic engineering strategies used.

Host Organism	Metabolic Engineering Strategy	Santalene Synthase	Redox System	Fermentation Scale	Yield	Ref.
*S. cerevisiae*	Altering ERG9 expression by replacing promoter with PHXT1; overexpression of santalol biosynthesis genes under GAL promotors.	SaSS and ClSS	CYP736A167-SaCPR2	Fed-batch, 5 L bioreactor	1.3 g/L santalol 1.2 g/L Z-α-santalol	[12]
*S. cerevisiae*	Integration of optimized P450-CPR redox system for santalols production; downregulating ERG9 gene.	SaSS	CYP736A167opt-46tATR1opt	100 mL shake flasks	164.7 mg/L santalene 68.8 mg/L santalol	[67]
*S. cerevisiae*	Optimization of precursor and cofactor supply; optimization of the FPP branch point; modulation of the MVA pathway; modification of the ammonium assimilation pathway; enhancing the activity of a transcriptional activator; continuous fermentation process optimization.	ClSS	-	0.3 L fermenter	0.036 Cmmol (g biomass)^−1^ h^−1^ of α-santalene	[88]
*Y. lipolytica*	Overexpression of MVA pathway genes; optimization of glucose concentration; fermentation optimization through genetic and feeding strategies.	ClSS	-	5-L fermenter	27.92 mg/L α-santalene	[91]
*E. coli*	Amplified flux toward FPP precursor; engineered santalene synthase through mutagenesis and fusion tag.	ClSS	-	100 mL shake flasks Fed-batch 1.3 L bioreactor	1272 mg/L α-santalene 2916 mg/L α-santalene	[68]
*E. coli*	Manipulated ribosome binding sites (RBSs) to optimize synthetic operon; deleted tnaA gene to increase α-santalene production	ClSS	-	100 mL shake flasks	599 mg/L α-santalene	[92]
*Komagataella phaffii* (*Pichia pastoris*)	Promoter optimization; gene overexpression; multi-copy integration for biosynthesis rewiring; medium optimization; bioprocess engineering.	SaSS	-	Shake flask Batch fermenter Fed-batch fermenter	829.8 mg/L α-santalene 4.4 g/L α-santalene 21.5 g/L α-santalene	[93]

## Data Availability

Not applicable.
